# MartiniGlass: a Tool for Enabling Visualization of Coarse-Grained Martini Topologies

**DOI:** 10.1021/acs.jcim.4c02277

**Published:** 2025-03-26

**Authors:** Christopher Brasnett, Siewert J. Marrink

**Affiliations:** † Groningen Biomolecular Sciences and Biotechnology Institute, University of Groningen, 9747 AG, Groningen, The Netherlands

## Abstract

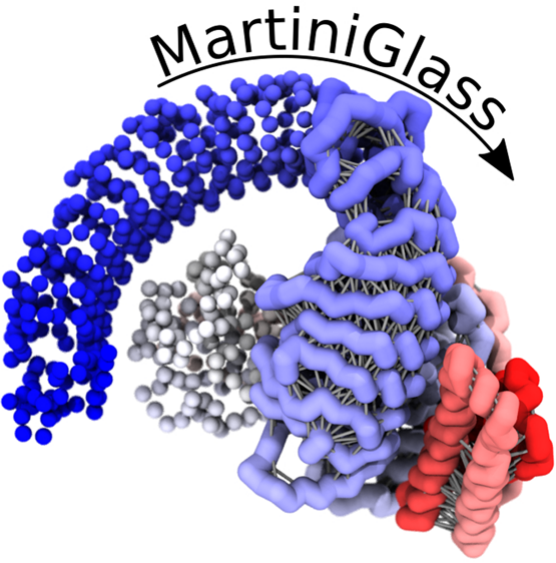

As molecular modeling gains ever more prominence in understanding cellular processes, high quality visualization of models and dynamics has never been more important. Naturally, much molecular visualization software is written to enable the visualization of atomic level details in structures. While necessary, this means that visualization of increasingly popular coarse-grained (CG) models remains a challenge. Here, we present a Python package, MartiniGlass, that facilitates the visualization of systems simulated with the widely used CG Martini force field using the popular visualization package VMD. MartiniGlass rapidly processes molecular topologies and accounts for important topological features at CG resolution, such as secondary structure restraints, preparing them for easy visualization of simulated trajectories.

## Introduction

**Figure 1:**
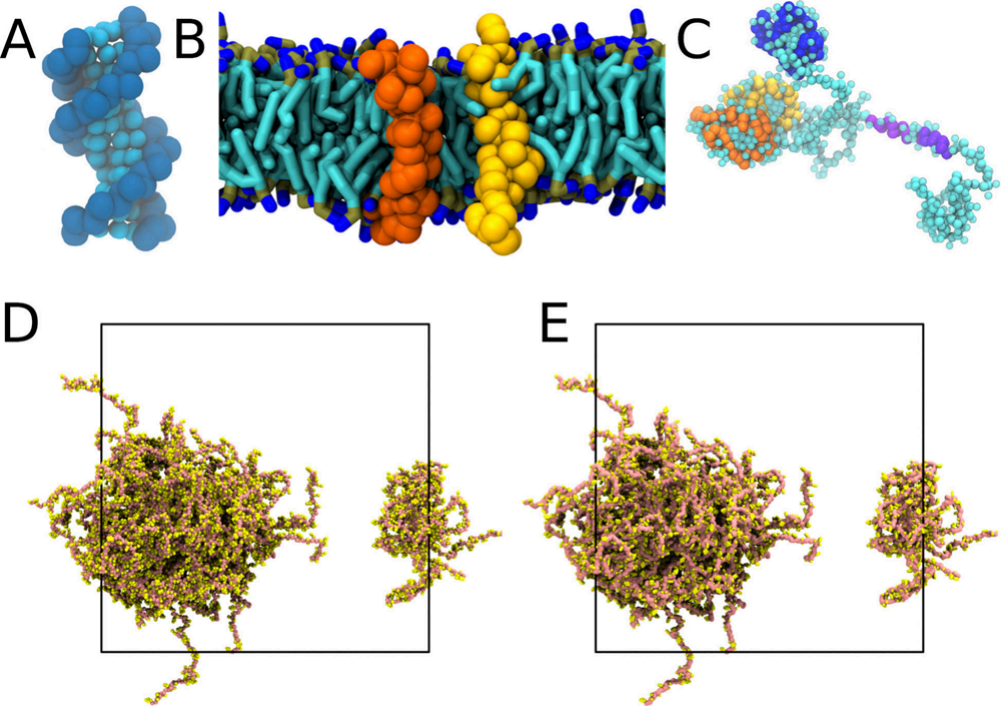
Examples of the visualization of Martini molecules. (A–C) Using spheres to represent coarse-grained beads: (A) DNA, (B) ErbB1 TM domains in a lipid bilayer, (C) a TDP-43 protein.^11−13^ (A) Reproduced with permission from Uusitalo et al. Copyright 2015 American Chemical Society.^13^ (B) Reproduced with permission from Javanainen et al. Copyright 2017 under creative commons attribution license.^12^ (C) Reproduced with permission from Ingólfsson et al. Copyright 2023 Biophysical Society under creative commons attribution license.^11^ (D, E) Condensate of proteins illustrated with (D) spheres and (E) licorice styles, demonstrating the cleaner appearance of a continuous style in dense, linked systems.

CG molecular dynamics simulations have significant advantages over higher resolution ones in accessing time scales and processes that are otherwise difficult to reach.^1,2^ However, in communicating the results of CG simulations, the use of standard simulation visualization software such as VMD^3^ can prove challenging. With atomistic structures, bonds can be drawn automatically between atoms based on either distance recognition or bonded information in structure files. Such automated methods are not possible at CG resolutions, as the apparent atoms (i.e., CG beads) in the system are necessarily further apart, and bonded information is not (usually) stored in trajectory files. Users can overcome this challenge by various means, whether by representing their molecules as a series of overlapping spheres to make it looked joined (Figure 1A–C), or in the case of CG proteins, by laborious editing of pdb files to ensure that CONECT records are written for the necessary bonds.

Here, we present a tool, MartiniGlass which facilitates better visualization of simulations performed with the popular Martini force field, using the VMD package.^2,4,5^ MartiniGlass was chosen as a name because without a glass, you can neither see nor drink a martini of your choice. The tool enables molecules to be visualized as continuous chains using the licorice or CPK styles of VMD (Figure 1D,E). MartiniGlass builds on previous work using a tcl script, cg_bonds, which had drawn both bonds and tertiary structure restraints by reading topology information from simulation files.^6^ Alternatively in the case of tertiary structure, the apparent bonds could also be illustrated using in-script calculation. In recent years, however, the use and maintenance of this script has proven increasingly challenging for two principal reasons. First, the format of Gromacs topology (tpr) files changes with each release, so reading topology information from the gmx dump tool requires regular updating. Second, many of the new models that have been developed for the latest version of Martini force field now make extensive use of topology constructs such as virtual sites, which are not trivial to read from topology information.^7−10^

## MartiniGlass

### General Functionality

MartiniGlass overcomes the issues of visualization discussed above by processing topology files for the system of interest. The processed topology files are intended for subsequent viewing together with structure and trajectory files in VMD. The processed topology files are derived from the bonded information present in the simulation input files, and pared down to exclude information not relevant for the purposes of visualization. Moreover, the program can flexibly handle topology features such as virtual sites, and makes it possible to also separately superimpose the tertiary structure restraints of proteins.

**Figure 2:**
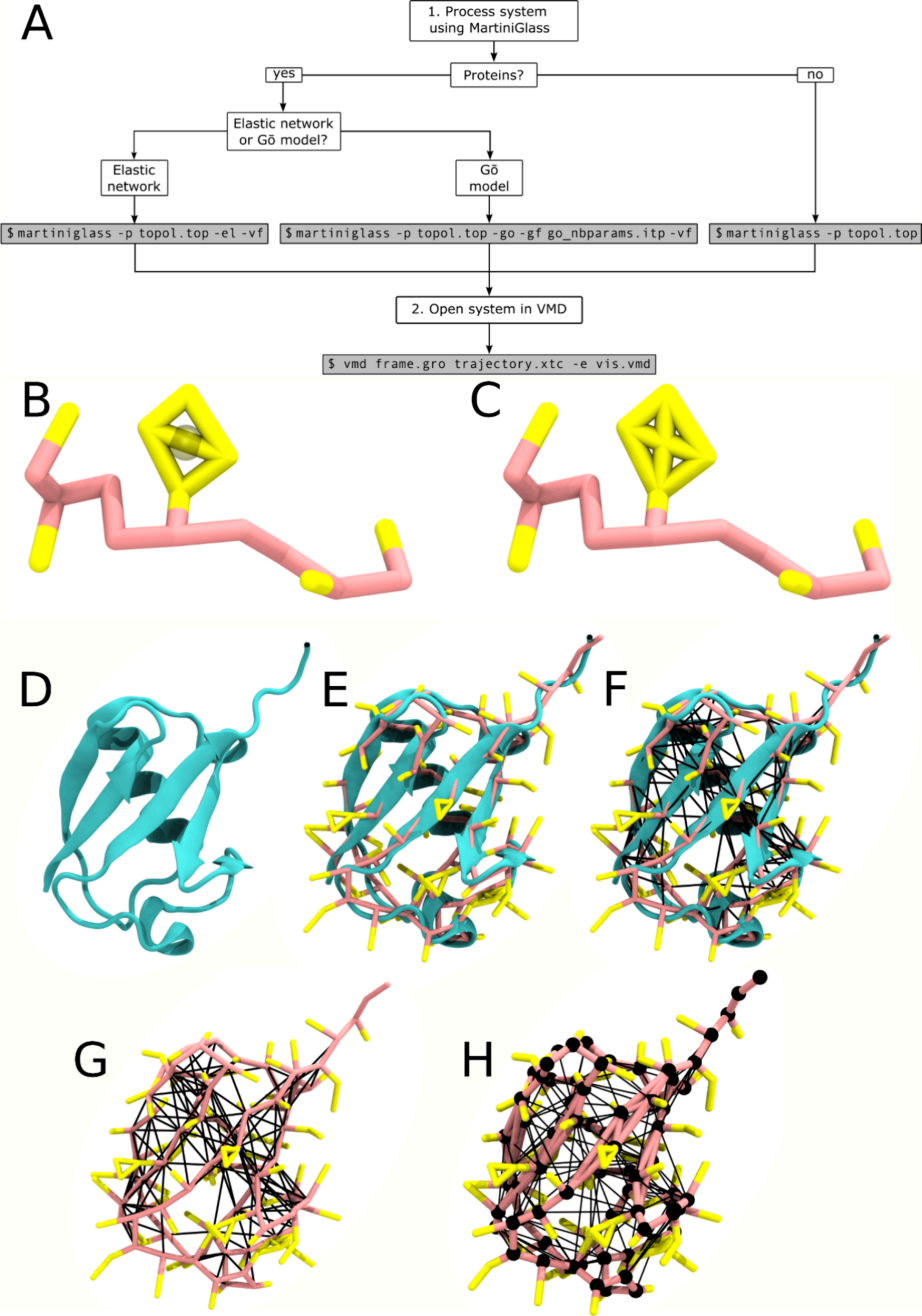
Visualization of CG proteins. (A) Flowchart of how the MartiniGlass program can be used to prepare systems for visualization in VMD. Boxes in gray indicate command line instructions. (B, C) Choosing how to illustrate virtual sites in a short peptide: (B) The virtual site of the tryptophan side chain is illustrated with a transparent bead, and all other bonds are illustrated as usual. (C) Using the default behavior of MartiniGlass, the virtual site has bonds drawn to its constructing atoms. (D–H) Ubiquitin (PDB code: 1UBQ) illustrated atomistically with secondary structure (D), the CG model overlaid (E), and with the elastic network (F). (G) The CG protein and elastic network on its own are shown, while (H) is the Go̅ model for the same protein, complete with the virtual backbone sites where the network arises from.

Topology processing is made possible by the use of the Vermouth library, primarily developed to power martinize2, but with further topology processing and manipulation functionalities.^14^ At its simplest, MartiniGlass reads a simulation system topology (.top) file, and processes all the included topology (.itp) files describing the molecules present in the system. After processing, the program writes out additional system and included topology files. The visualization topologies are intended to be subsequently loaded onto structure files opened in VMD via the cg_bonds tcl script. The automation of this process is enabled by the provision of several VMD state files provided with the MartiniGlass program. The provided state files automatically load the cg_bonds program, and the visualization topologies associated with the system. Viewing in VMD is still fundamentally facilitated by the cg_bonds tcl script, the latest version of which has been optimized for use in conjunction with MartiniGlass. For ease of reference, both the cg_bonds and an associated VMD visualization state can be written locally when MartiniGlass is executed. A flowchart of how the program is used with some example commands is shown in Figure 2A. The examples shown all make use of the -vf flag, which writes VMD state files for users to use as additional inputs to VMD to view their systems.

Visualization topologies contain only three Gromacs directives: the molecule type, atoms, and bonds.^4^ As no other topology information is required for visualization, they are removed from the molecule description. However, this requires decisions to be made about how some of the challenges discussed above are to be addressed. In the case of virtual sites, users are able to decide whether virtual sites should have bonds drawn between them and all their constructing atoms, or left as free-floating, which in certain cases may be more desirable (Figure 2B,C).

### Application to Protein Systems

As well as direct bonds between contiguous beads, Martini protein models also use a variety of additional restraints to maintain protein structure throughout simulation.^15−17^ The additional restraints are in the form of either harmonic bonds (in the case of elastic networks), or site-specific nonbonded potentials (as in the case of the Go̅ or OLIVES models).

Understanding the restraints that these networks put on simulation input models, and their physical relevance to the underlying protein structure can be an important challenge in optimizing models. For example, the initial input structure to a program such as martinize2 (which generates Martini protein topologies) may result in elastic network bonds being drawn between folded and disordered domains of a protein, because they happen to be close in a starting structure.^14^ In reality - and in atomistic simulations - the two domains would likely move apart after a short space of time and no such bond should be generated in the first place, highlighting the need for visual checks during optimization.

Drawing these networks then is an important consideration in the visualization of coarse-grained Martini proteins, but is also a further challenge in visualization. The so-called bonds in these networks have (i) significantly longer lengths, and (ii) are explicitly not drawn between numerically adjacent atoms from the structure file, and are therefore only included in the topology information. The recent development of the MArtini Database Server (MAD) for interactive generation of Martini proteins has gone some way to helping users refine the initial topologies generated by martinize2.^18^ MAD allows users to interact with the generated elastic network through a visualization facilitated by the online NGL Viewer package, so users can choose, add or delete elastic network bonds as they require.^19^ Externally to the MAD, NGL viewer offers some automatic detection of CG bonds, but has drawbacks in necessarily being used via online notebooks, and does not easily facilitate viewing of secondary structure networks.

The most significant feature that MartiniGlass enables in topology handling is the distinction of tertiary structure networks for protein models (Figure 2C–G). If the requisite process is specified, MartiniGlass will generate separate topology files for the direct bonded network of a protein, and its tertiary structure nonbonded interactions (in the case of Go̅ or OLIVES models) or harmonic bonds (in the case of elastic networks). Where the tertiary structure is maintained by specific nonbonded interactions, nonbonded interactions are read from the associated file and converted to bonds in an input topology file distinct from the direct bonded network of the protein.

For elastic networks, elastic bonds are identified as bonds between backbone beads in nonconsecutive residues. Once these bonds have been identified, MartiniGlass will perform additional checks on the elastic network to ensure that the model can be viewed in VMD. VMD has an upper limit of 12 on the possible number of bonds an atom can have. As elastic networks generated by martinize2 are drawn arbitrarily by a distance cut off, it is possible for backbone atoms to have more than 12 bonds, causing a fault in the visualization. In cases where more than 12 bonds are found, the network is edited to ensure that apparent excess bonds are removed and noted, enabling straightforward visualization of the elastic network in VMD. As tertiary structure bond networks are written separately to the principle topology of the protein, they can be loaded into VMD separately, and switched on and off in visualizations as required.

**Figure 3:**
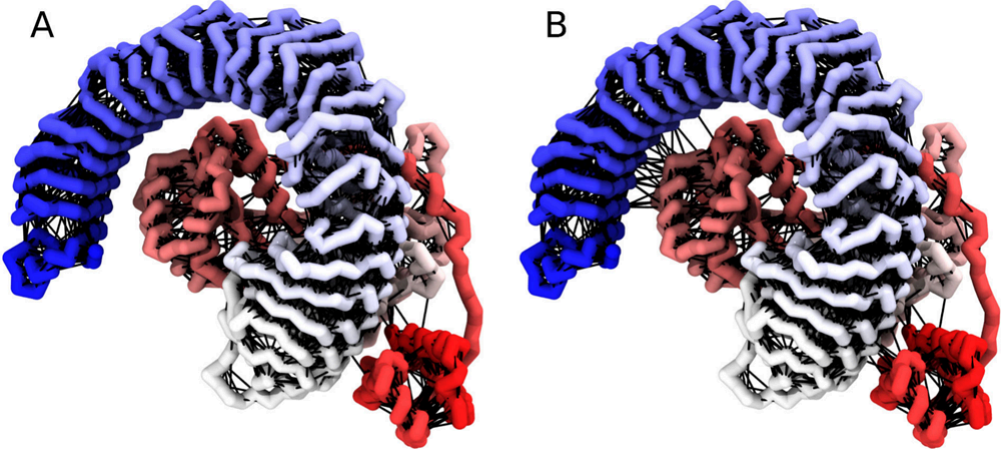
Different methods of visualizing elastic bonds. (A) The default elastic network for viewing in VMD as processed by MartiniGlass. (B) The full elastic network of the molecule.

While the edited network enables complex elastic networks to be visualized dynamically, this is not always desirable, as the editing of bonds may mean that once the structure is checked visually, undesirable bonds are missed. To enable full checking of the elastic network, the network can also be written as an additional script to be read by VMD to draw static cylinders in place of bonds. As this method is not limited by any topology information within VMD, the full network can be drawn. However, as the cylinders drawn are static they do not update with trajectory information, and are only intended for single structure information. We highlight such an example in Figure 3 with Probable disease resistance protein At1g58602 (structure AF-Q8W3K0-F1-v4), where the edited network (Figure 3A) has some interdomain bonds missing in comparison to the full network (Figure 3B).

**Figure 4:**
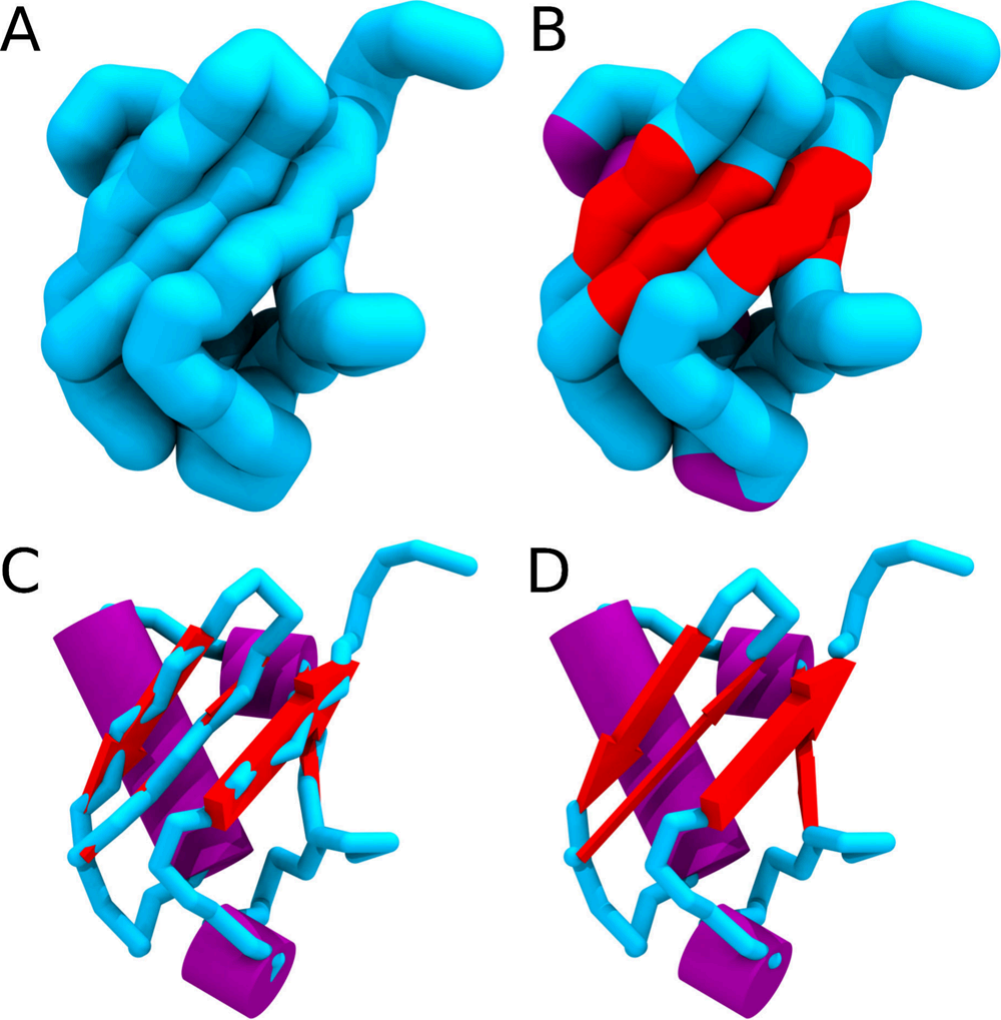
Visualizing secondary structure elements of a protein. (A) UBQ1 shown as a continuous backbone ribbon. (B) Colored by secondary structure (helices in purple, sheets in red). (C) Arrows (for sheets) and cylinders (for helices) overlaid on a backbone ribbon. (D) As in (C), but with the backbone removed from underneath the overlaid geometry objects.

A further challenge for the visualization of coarse grained proteins is indicating their secondary structure. This was also facilitated by a second tcl script to draw basic geometric objects in the place of secondary structure elements. Examples of how this information can be visualized are shown in Figure 4, either through the additional overlay of objects representing secondary structure, or by recolouring parts of the backbone of the protein. When protein topologies with secondary structure information are provided to MartiniGlass, information about how to conduct such visualizations are written for users to use as they wish.

## Conclusions

**Figure 5:**
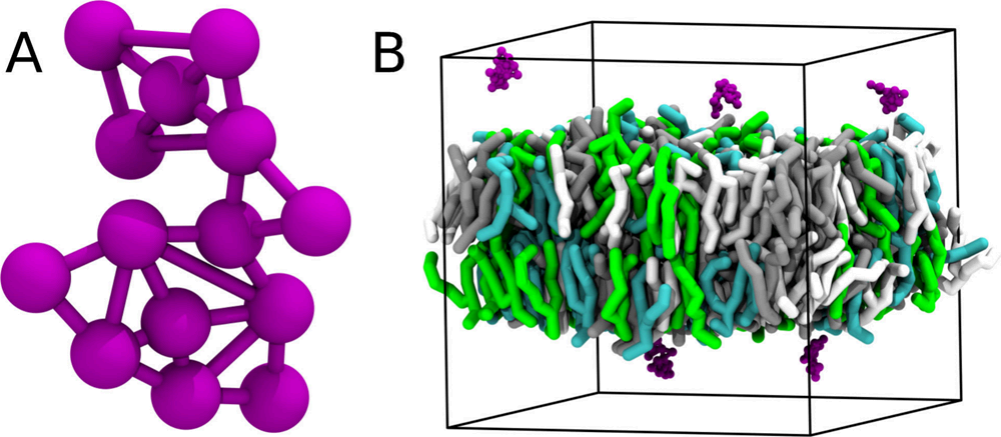
Nonprotein systems. (A) The molecular motor of Vainikka and Marrink, a synthetic molecule with complex topology, showing how the beads are connected.^8^ (B) Several molecular motors together with a lipid bilayer of several different lipid types.

Here, we have presented an overview of a new tool, MartiniGlass, which makes it possible to better visualize CG molecules from the Martini force field and their simulations. A selection of how MartiniGlass may be used to generate visualizable topology files is shown in Table 1. While MartiniGlass takes extensive care over how tertiary structure networks in proteins are handled to ensure that their visualization is both possible and flexible, it is really built to handle any kind of molecule parametrized with the Martini force field, from proteins and DNA, lipids and sugars, through to deep eutectic solvents and molecular motors, to ensure that their bonded connections are truly visible. Although much of the above has shown how MartiniGlass is particularly useful for the visualization of proteins, we further show in Figure 5 that the tool is applicable to generic and complex systems. Here we have focused on an application for molecules in the Martini force field, but there is no reason why in future this method could not be expanded to other CG force fields that use Gromacs. Similarly, while MartiniGlass has been particularly designed for visualizing CG simulations with VMD, its functionality allows for bond atom indices to be written to text file to read into other visualization software such as pymol or ngl viewer.^20^

**Table 1:** Example Commands for MartiniGlass

description	command
Basic execution	MartiniGlass -p topol.top
Output a nonwater index file for trajectory processing	MartiniGlass -p topol.top -f frame.gro
Do not write bonds between virtual sites and their constructing atoms	MartiniGlass -p topol.top -vs
Process protein elastic networks into separate visualization topologies	MartiniGlass -p topol.top -el
Process Go̅ networks in proteins, as defined in nonbond.itp into separate visualization topologies	MartiniGlass -p topol.top -go -gf nonbond.itp
Write out visualization state files (cg_bonds-v6.tcl, vis.vmd) in the current directory	MartiniGlass -p topol.top -vf
Write out the full elastic network of a protein that has >12 bonds on some atoms as a .tcl file to draw static cylinders in place of dynamic bonds	MartiniGlass -p topol.top -cyl -f frame.gro -el

## Data Availability

The code is available on GitHub https://github.com/Martini-Force-Field-Initiative/MartiniGlass and is installable with pip. Full documentation complete with worked examples of how the package can be used are available at https://martiniglass.readthedocs.io/en/latest.

## References

[ref1] Borges-Araújo L.; Patmanidis I.; Singh A. P.; Santos L. H. S.; Sieradzan A. K.; Vanni S.; Czaplewski C.; Pantano S.; Shinoda W.; Monticelli L.; Liwo A.; Marrink S. J.; Souza P. C. T. Pragmatic Coarse-Graining of Proteins: Models and Applications. J. Chem. Theory Comput. 2023, 19, 7112 10.1021/acs.jctc.3c00733. 37788237

[ref2] Souza P. C. T.; Alessandri R.; Barnoud J.; Thallmair S.; Faustino I.; Grünewald F.; Patmanidis I.; Abdizadeh H.; Bruininks B. M. H.; Wassenaar T. A.; Kroon P. C.; Melcr J.; Nieto V.; Corradi V.; Khan H. M.; Domański J.; Javanainen M.; Martinez-Seara H.; Reuter N.; Best R. B.; Vattulainen I.; Monticelli L.; Periole X.; Tieleman D. P.; de Vries A. H.; Marrink S. J. Martini 3: A General Purpose Force Field for Coarse-Grained Molecular Dynamics. Nat. Methods 2021, 18 (4), 382–388. 10.1038/s41592-021-01098-3. 33782607 PMC12554258

[ref3] Humphrey W.; Dalke A.; Schulten K. VMD: Visual Molecular Dynamics. J. Mol. Graph. 1996, 14 (1), 33–38. 10.1016/0263-7855(96)00018-5. 8744570

[ref4] Abraham M. J.; Murtola T.; Schulz R.; Páll S.; Smith J. C.; Hess B.; Lindahl E. GROMACS: High Performance Molecular Simulations through Multi-Level Parallelism from Laptops to Supercomputers. SoftwareX 2015, 1–2, 19–25. 10.1016/j.softx.2015.06.001.

[ref5] Marrink S. J.; Monticelli L.; Melo M. N.; Alessandri R.; Tieleman D. P.; Souza P. C. T. Two Decades of Martini: Better Beads, Broader Scope. WIREs Comput. Mol. Sci. 2023, 13 (1), e1620 10.1002/wcms.1620.

[ref6] CG bonds. Martini Force Field Initiative. https://cgmartini.nl/docs/downloads/tools/visualization.html (accessed 2024–08–28).

[ref7] Borges-Araújo L.; Borges-Araújo A. C.; Ozturk T. N.; Ramirez-Echemendia D. P.; Fábián B.; Carpenter T. S.; Thallmair S.; Barnoud J.; Ingólfsson H. I.; Hummer G.; Tieleman D. P.; Marrink S. J.; Souza P. C. T.; Melo M. N. Martini 3 Coarse-Grained Force Field for Cholesterol. J. Chem. Theory Comput. 2023, 19 (20), 7387–7404. 10.1021/acs.jctc.3c00547. 37796943

[ref8] Vainikka P.; Marrink S. J. Martini 3 Coarse-Grained Model for Second-Generation Unidirectional Molecular Motors and Switches. J. Chem. Theory Comput. 2023, 19 (2), 596–604. 10.1021/acs.jctc.2c00796. 36625495 PMC9878727

[ref9] Vainikka P.; Thallmair S.; Souza P. C. T.; Marrink S. J. Martini 3 Coarse-Grained Model for Type III Deep Eutectic Solvents: Thermodynamic, Structural, and Extraction Properties. ACS Sustain. Chem. Eng. 2021, 9 (51), 17338–17350. 10.1021/acssuschemeng.1c06521.

[ref10] Grünewald F.; Punt M. H.; Jefferys E. E.; Vainikka P. A.; König M.; Virtanen V.; Meyer T. A.; Pezeshkian W.; Gormley A. J.; Karonen M.; Sansom M. S. P.; Souza P. C. T.; Marrink S. J. Martini 3 Coarse-Grained Force Field for Carbohydrates. J. Chem. Theory Comput. 2022, 18 (12), 7555–7569. 10.1021/acs.jctc.2c00757. 36342474 PMC9753587

[ref11] Ingólfsson H. I.; Rizuan A.; Liu X.; Mohanty P.; Souza P. C. T.; Marrink S. J.; Bowers M. T.; Mittal J.; Berry J. Multiscale Simulations Reveal TDP-43 Molecular-Level Interactions Driving Condensation. Biophys. J. 2023, 122 (22), 4370–4381. 10.1016/j.bpj.2023.10.016. 37853696 PMC10720261

[ref12] Javanainen M.; Martinez-Seara H.; Vattulainen I. Excessive Aggregation of Membrane Proteins in the Martini Model. PLoS One 2017, 12 (11), e0187936 10.1371/journal.pone.0187936. 29131844 PMC5683612

[ref13] Uusitalo J. J.; Ingólfsson H. I.; Akhshi P.; Tieleman D. P.; Marrink S. J. Martini Coarse-Grained Force Field: Extension to DNA. J. Chem. Theory Comput. 2015, 11 (8), 3932–3945. 10.1021/acs.jctc.5b00286. 26574472

[ref14] Kroon P. C.; Grünewald F.; Barnoud J.; van Tilburg M.; Souza P. C. T.; Wassenaar T. A.; Marrink S.-J. Martinize2 and Vermouth: Unified Framework for Topology Generation. eLife 12, RP90627 2023, 10.48550/arXiv.2212.01191. PMC12634043 41263305

[ref15] Souza P. C. T.; Araujo L. P. B.; Brasnett C.; Moreira R. A.; Grunewald F.; Park P.; Wang L.; Razmazma H.; Borges-Araujo A. C.; Cofas-Vargas L. F.; Monticelli L.; Mera-Adasme R.; Melo M. N.; Wu S.; Marrink S. J.; Poma A. B.; Thallmair S. Go̅Martini 3: From Large Conformational Changes in Proteins to Environmental Bias Corrections. bioRxiv 2024, 10.1101/2024.04.15.589479. PMC12043922 40307210

[ref16] Pedersen K. B.; Borges-Araújo L.; Stange A. D.; Souza P. C. T.; Marrink S. J.; Schiøtt B. OLIVES: A Go̅-like Model for Stabilizing Protein Structure via Hydrogen Bonding Native Contacts in the Martini 3 Coarse-Grained Force Field. J. Chem. Theory Comput. 2024, 10.1021/acs.jctc.4c00553. 39235392

[ref17] Periole X.; Cavalli M.; Marrink S.-J.; Ceruso M. A. Combining an Elastic Network With a Coarse-Grained Molecular Force Field: Structure, Dynamics, and Intermolecular Recognition. J. Chem. Theory Comput. 2009, 5 (9), 2531–2543. 10.1021/ct9002114. 26616630

[ref18] Hilpert C.; Beranger L.; Souza P. C. T.; Vainikka P. A.; Nieto V.; Marrink S. J.; Monticelli L.; Launay G. Facilitating CG Simulations with MAD: The MArtini Database Server. J. Chem. Inf. Model. 2023, 63 (3), 702–710. 10.1021/acs.jcim.2c01375. 36656159

[ref19] Rose A. S.; Hildebrand P. W. NGL Viewer: A Web Application for Molecular Visualization. Nucleic Acids Res. 2015, 43 (W1), W576–W579. 10.1093/nar/gkv402. 25925569 PMC4489237

[ref20] Schrödinger, LLC. The PyMOL Molecular Graphics System, Version 1.8, 2015.

